# Erosive pustular dermatosis of the lower legs (EPDL): A rarely diagnosed neutrophilic dermatosis of the elderly

**DOI:** 10.1111/ddg.70071x

**Published:** 2026-02-28

**Authors:** Joachim Dissemond, Jan Kottner, Jan‐Malte Placke, Cornelia Erfurt‐Berge

**Affiliations:** ^1^ Department of Dermatology Venereology and Allergology University Medical Center Essen Essen Germany; ^2^ Charité‐Universitätsmedizin Berlin Institute of Clinical Nursing Science Berlin Germany; ^3^ Department of Dermatology University Medical Center Erlangen Erlangen Germany

**Keywords:** atrophy, chronic venous insufficiency, compression therapy, neutrophilic dermatosis, pustules

## Abstract

Erosive pustular dermatosis of the lower legs (EPDL) is a rarely diagnosed, chronic inflammatory skin disease that occurs predominantly in elderly people. Predisposing factors include skin atrophy, chronic venous insufficiency, and trauma. Although the pathogenesis of EPDL has not yet been conclusively clarified, there are ongoing discussions on its nature as a neutrophilic dermatosis in which exogenous triggers lead to immunological dysregulation with local skin damage. Clinically, EPDL manifests with superficial, sterile pustules from which sharply defined erosions develop. The predilection sites are the middle third of the lower leg extensor sides. It is a diagnosis of exclusion, which makes diagnostic differentiation more difficult. Potent topical glucocorticoids with a high therapeutic index (TIX) and calcineurin inhibitors are used therapeutically. Systemic immunomodulating therapies are reserved for refractory courses. Optimization of wound care is also important. In the long term, education and skin care are at the forefront of the complex and often long‐term treatment. Due to the chronic recurrent course and the risk of secondary ulcerations and superinfections, early diagnosis and individual treatment planning is important. Interdisciplinary and interprofessional collaboration can make a decisive contribution to improve quality of life of affected patients and reduce the risk of complications.

## INTRODUCTION

*Erosive pustular dermatosis of the scalp* (EPDS) was first described by Burton in 1977.^1^ In 1987, Lanigan and Cotteril published a clinically similar variant on the lower legs that occurs predominantly in elderly people in atrophic and/or actinically damaged skin.^2^ This dermatosis is referred to as *erosive pustular dermatosis of the leg* (EPDL) and was so far only rarely a subject of discussion in the scientific literature. Accordingly, the disease is often overlooked or misdiagnosed, and reliable epidemiological data on prevalence and incidence are therefore lacking.^3^

## CLINICAL APPEARANCE

**FIGURE 1:**
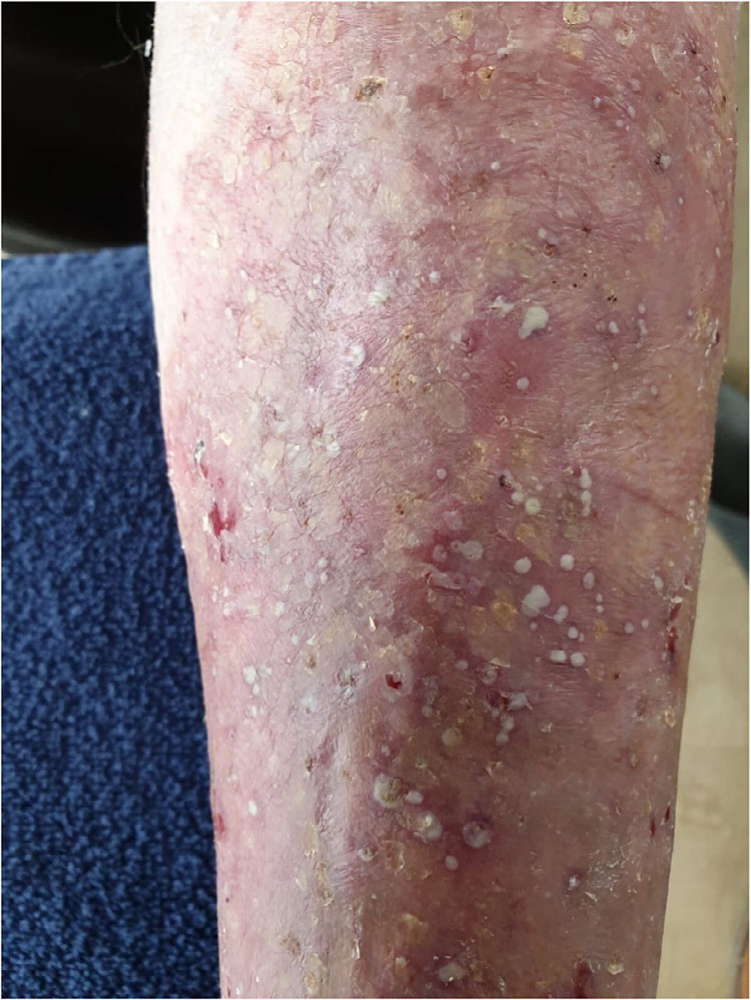
Sterile pustules in a patient with CVI and compression therapy. The diagnosis of EPDL was subsequently made.

**FIGURE 2:**
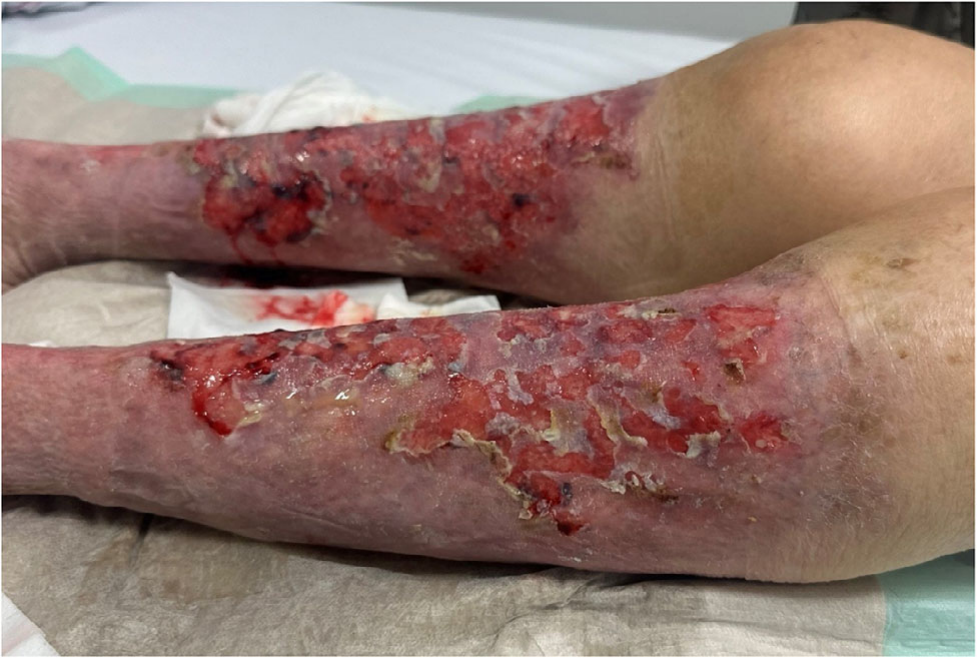
The border of the acute erosions in EPDL is sharp and serpiginous. A collerette‐like scaling can often be seen at the edge.

**FIGURE 3:**
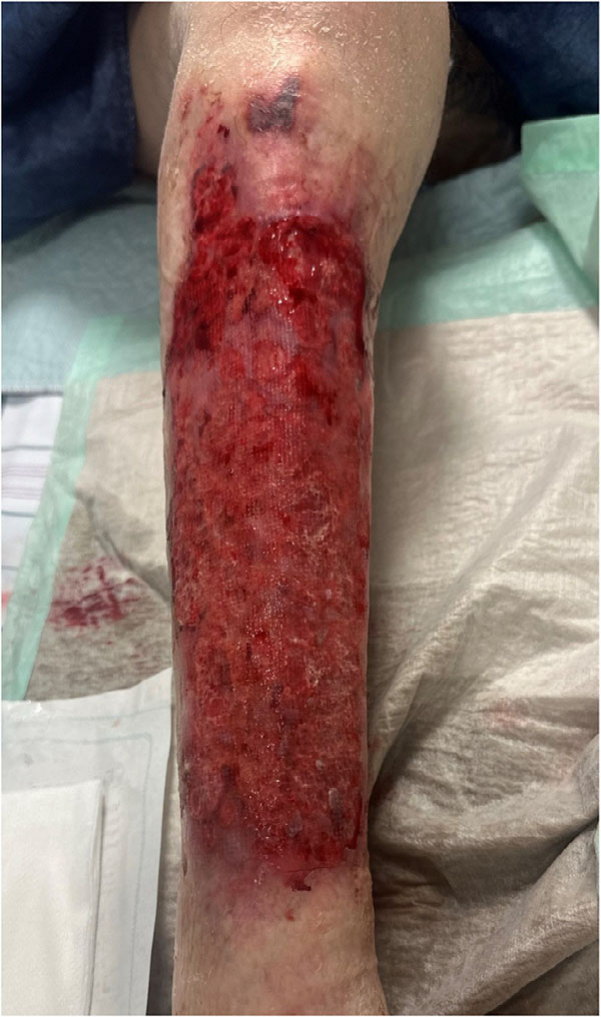
The persistent erosions are sharply limited to the area covered by the wound dressing.

Skin lesions typically manifest with multiple superficial pustules a few millimeters in size (Figure 1) from which large erosions with serpiginous edge and, in part, collerette‐like scaling develop rapidly (Figure 2). Subsequently, a sharply defined erosion persists (Figure 3). Secondary bacterial and, sometimes, fungal superinfections and crusts may form on top of the erosions,^4^, ^5^ even though the pustules are primarily sterile. The skin lesions occur predominantly on ventral or anteromedial aspects of the lower legs and are often localized on both legs. Usually, affected patients report pruritus, burning sensation, and mild pain.^4^, ^6^

## DERMATOPATHOLOGY

**FIGURE 4:**
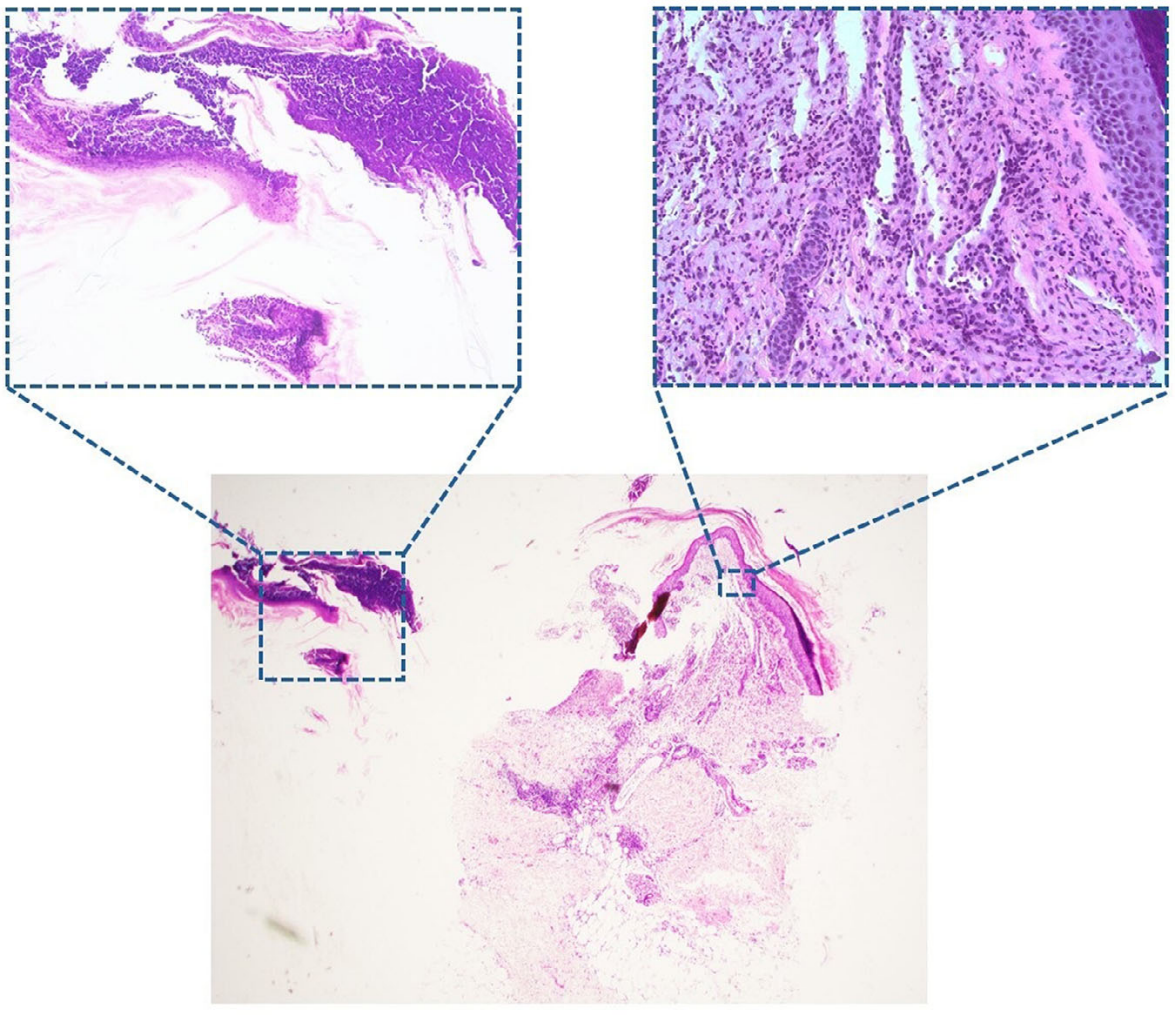
The histopathological overview shows hyperparakeratosis and neutrophilic granulocytes in the stratum corneum. Edema and mixed‐cell, neutrophil‐rich inflammation are visible in the upper dermis. The magnification at the top left shows the stratum corneum with numerous neutrophil granulocytes, while the magnification on the right illustrates the dense, neutrophil‐rich inflammatory infiltrate in the dermis (hematoxylin‐eosin staining).

If the diagnosis is uncertain, a biopsy should be taken for histopathological workup. Histopathology typically shows subcorneal or intracorneal pustules with superficial mixed‐cell infiltration by neutrophilic granulocytes and plasma cells as well as epidermal atrophy (Figure 4).^5^, ^7^

Given that the histopathological presentation is also nonspecific, EPDL is diagnosed by exclusion of other diseases.^3^ Accordingly, the relevant differential diagnoses must be known and excluded (Table 1).

**TABLE 1:** Clinically relevant differential diagnoses of EPDL.

Acute localized exanthematous pustulosis (ALEP) Autoimmune bullous dermatoses, for example, bullous pemphigoid Contact dermatitis Pustular psoriasis Pyoderma gangrenosum Pyodermas like impetigo, erysipelas Stasis dermatitis

## PATHOGENESIS

The exact etiopathogenesis of EPDL is currently unclear. Various predisposing local and systemic factors have been described (Table 2). Analogous to EPDS, an association with drugs, such as inhibitors of EGFR (*epidermal growth factor* receptor) tyrosine kinase, may be discussed, as well.^8^ It has been described that EPDL, similar to other neutrophilic dermatoses, may be triggered by release of DAMPs (*damage‐associated molecular patterns*) due to trauma or of PAMPs (*pathogen‐associated molecular patterns*) due to microorganisms. This induces a dysregulation of the innate immune system with abnormal activation of neutrophilic granulocytes.^9^, ^10^ However, courses with spontaneous manifestation have also been described.^11^

**TABLE 2:** Predisposing factors of EPDL.

Actinically damaged skin Atopic diathesis Chronic venous insufficiency Diabetes mellitus Eczemas Skin atrophy, for example, due to glucocorticoids or ionizing radiation Advanced age Malnutrition, for example, zinc deficiency Maceration, especially under wound dressings Edemas Traumas, for example, due to dressings, care products, or falls Xerosis cutis

## THERAPY

Successful treatments are based on topical application of potent glucocorticoids.^2^, ^7^ Because of the often pronounced skin atrophy, only topical glucocorticoids with high therapeutic index (TIX), such as mometasone furoate, methylprednisolone aceponate, or prednicarbate, each with a TIX of 2.0, should be used, if possible.^12^ Especially for long‐term local therapy, the calcineurin inhibitors tacrolimus or pimecrolimus may also be used in order to prevent a *rebound* phenomenon after discontinuation of glucocorticoids.^13^ From a pathophysiological view, topical Janus kinase inhibitors (JAKi) like ruxolitinib and delgocitinib without atrophogenic potential might be used in future.^14^ However, clinical studies on these agents are currently lacking. While the galenic principles should be considered when selecting individually suitable substances, care must be taken to ensure that the topicals are applied as atraumatic as possible. Antiseptic treatment (for example, with polyhexanide) is useful in case of bacterial superinfection.^15^ Systemic immunomodulating therapy with glucocorticoids or dapsone should be reserved for very pronounced and refractory courses (Table 3).

**TABLE 3:** Therapy algorithm of EPDL.

Therapy	Agent	Notes
**1. choice** **(topical)**	Potent glucocorticoids Mometasone furoate Methylprednisolone aceponate Prednicarbate	TIX ≥ 2.0 recommended
**Long‐term therapy**	Calcineurin inhibitors Tacrolimus Pimecrolimus	Prevention of rebound phenomenon after glucocorticoids
**Future options** **(experimental)**	Topical Janus kinase inhibitors (JAKi) Ruxolitinib Delgocitinib	As yet, insufficient clinical data
**Superinfection**	Antiseptics Polyhexanide Octenidine	Adjunctive therapy in case of microbial superinfection
**Systemic therapy**	Glucocorticoids (prednisone/prednisolone) Dapsone	In very severe and/or refractory courses

It is important to avoid known risk factors as much as possible. A practical problem arises in patients with chronic venous insufficiency (CVI) and mechanical irritation due to compression therapy. Medically adaptive compression systems (MAC) are less traumatic for the skin while ensuring the compression therapy urgently recommended for medical reasons in CVI and may present an alternative in this context.^16^ Especially under occlusive and/or adhesive wound dressings a moist and warm environment is formed. Especially in case of long‐term use or heavy perspiration, this may result in macerations and irritations or (micro)trauma. Accordingly, the dressing strategy should be reconsidered and adjusted in affected patients. Particularly suitable are non‐adhesive, preferably breathable wound products that ensure good exudate management. Moreover, it is recommended to perform dressing changes in shorter intervals, possibly even daily.^17^

Usually, EPDL has a chronic recurrent course. If adequately treated, it may heal without ulceration, but the skin remains atrophic and susceptible to recurrence. If the disease is left untreated or insufficiently treated, there is a risk of ulceration, bacterial superinfection, and, in rare cases, even squamous cell carcinomas on chronically damaged skin.^18^ Apart from strict skin care, education of affected patients or relatives and nursing services is important in the long term to raise awareness for this disease entity with interdisciplinary and interprofessional relevance.^19^

## DISCUSSION

The currently very limited level of knowledge on EPDL often results in misdiagnoses and inadequate treatment approaches causing unnecessary delays of the disease course and significant impairment of the quality of life of affected patients. As yet, there are no controlled clinical trials addressing this disease. The few available publications are mostly case reports or case series. In a French multicenter study, the data of 36 patients with EPDL from 13 treatment centers were evaluated. The mean age was 79.6 years. Men were approximately five times more frequently affected than women; 91.7 % of the patients had CVI. In 53 % of the cases, the skin lesions occurred bilaterally; the ventral middle third of the lower legs was identified as the most common predilection site. Complete healing was observed in almost 80 % of affected patients after an average of 2.5 months. In 97.3 % of the cases, topical glucocorticoids were used for treatment. Approximately 40 % of the patients experienced recurrence after a mean follow‐up period of 2.5 months.^5^ In another study, pustules on the legs were documented in 24 of 400 patients with advanced CVI. Fungal infection was identified in 13 of these 24 patients. The authors discussed that EPDL is more common in patients with CVI undergoing compression therapy, especially in case of fungal infection.^4^ In a retrospective analysis of 16 patients with a mean age of 81 years, EPDL affected also approximately five times more men than women. Again, predilection sites were the middle thirds of the ventral aspect of the lower legs, and in 63 % of the patients the disease occurred bilaterally. Clinically, ochre dermatitis and skin atrophy was described in most cases. Apart from CVI, the authors also discussed UV‐related skin atrophy as potentially contributing factor. In 12 of 13 cases, topical glucocorticoids were effective with a mean treatment duration of six months. Subsequently, recurrence occurred in 50 % of patients.^18^ In Italy, 51 patients with a mean age of 81 years were documented in a case collection over a period of ten years. The gender distribution was roughly balanced. In 43 % of the affected patients the skin lesions occurred bilaterally. In 17 of the 40 patients assessed for atopic diathesis, this disorder was confirmed.^6^ Based on the examination of three patients with EPDL, it was discussed that substitution of zinc gluconate might be useful, given that zinc deficiency was identified in these patients.^20^

EPDL may be included in the group of neutrophilic dermatoses. Neutrophilic dermatoses are a heterogeneous group of non‐infectious inflammatory skin diseases histopathologically characterized by sterile infiltration of the skin with neutrophilic granulocytes. They result from dysregulation of the innate and, in part, adaptive immune system causing excessive activation and recruitment of neutrophilic granulocytes via a cascade of proinflammatory cytokines and activating inflammasomes. Neutrophilic dermatoses can cause diverse cutaneous and extracutaneous manifestations and are, therefore, associated with significant morbidity and mortality.^10^, ^21^ They may occur idiopathically or in systemic diseases, such as autoimmune diseases, neoplasms, or infections, or as partial symptom of autoinflammatory syndromes, such as PAPA (pyogenic arthritis, pyoderma gangrenosum, acne), PASH (pyoderma gangrenosum, acne, hidradenitis suppurativa), DIRA (deficiency of interleukin‐1 receptor antagonist), or CAPS (cryopyrin‐associated periodic syndromes).^22^ Accordingly, a further diagnostic workup, such as white blood cell differential and instrumental imaging, should always be performed in these patients based on their medical history and the clinical findings.^8^, ^23^, ^24^ In the initial stages, the clinical features of EPDL and pyoderma gangrenosum (PG) are often similar, given that both dermatoses may initially manifest with sterile pustules on the extensor sides of the lower legs. Taking a biopsy is here often useful, given that, in contrast to EPDL, PG shows a significantly deeper and denser infiltrate with neutrophilic granulocytes and usually also an associated leukocytoclastic vasculitis. In contrast to EPDL, PG is, therefore, almost always associated with severe pain and a dark‐livid rim.^10^ If present, superficial (subcorneal) localized pustuloses are currently classified by various terms, such as acute localized exanthematous pustulosis (ALEP).^25^, ^26^ A pathophysiological association with (paradoxical) drug reactions is usually discussed in this context. In daily clinical practice, there are always patients whose disease cannot be assigned conclusively to a certain neutrophilic dermatosis. However, for the affected patients, especially the identification of relevant trigger factors, targeted therapy, and, if possible, preventive measures are of crucial importance.

Based on the characteristic clinical presentation, the comparable histopathological findings, and the evidence of underlying pathophysiological mechanisms, we suggest the future classification of EPDL as neutrophilic dermatosis.

## CONCLUSION

EPDL is an idiopathic inflammatory dermatosis with special topographical manifestation. Although rarely diagnosed, it is an important disease especially in elderly people and, in particular, in patients with CVI. Given the similarity with infectious or autoimmune diseases, thorough differential diagnosis is required. While the complex therapies are usually very effective, they require a strict skin care regimen and long‐term management in an interdisciplinary and interprofessional team.

## CONFLICT OF INTEREST STATEMENT

None.
